# Development and application of a rapid visual detection technique for VanA gene in vancomycin-resistant *Enterococcus faecium*

**DOI:** 10.1128/msphere.00666-24

**Published:** 2024-09-10

**Authors:** Tuo Ji, Wenjun Wang, Li Wang, Yuzhi Gao, Yan Wang, Xuzhu Gao

**Affiliations:** 1 Institute of Clinical Oncology, Lianyungang Hospital Affiliated to Kangda College of Nanjing Medical University, Lianyungang, China; 2 Department of Central Laboratory, The Second People’s Hospital of Lianyungang (Cancer Hospital of Lianyungang), Lianyungang, China; Nanjing University of Chinese Medicine, Nanjing, Jiangsu, China

**Keywords:** multienzyme isothermal rapid amplification, lateral flow strips, vancomycin-resistant, *Enterococcus faecium*

## Abstract

The objective of this study was to establish a rapid visual diagnosis method for vancomycin-resistant *Enterococcus faecium* (VREFm) based on multienzyme isothermal rapid amplification (MIRA) combined with lateral flow strips (LFSs). The MIRA primers and probes were specifically designed to maintain the sequence of the VanA gene of VREFm. We optimized the reaction time and temperature and thoroughly assessed the specificity and sensitivity of the MIRA-LFS system. We also compared the MIRA-LFS method with the polymerase chain reaction (PCR) assay and the disc diffusion method. We then evaluated the MIRA-LFS assay for consistency testing and clinical application. The MIRA-LFS technique completed the amplification process within 30 min, and the results were observed on LFS. The method demonstrated high sensitivity, with a minimum detection limit of 1.066 CFU/µL for VREFm and exhibited specificity without cross-reactivity with other pathogenic bacteria. When applied to the detection of clinical samples, the method exhibited consistency with the PCR and agar dilution methods. The combined use of MIRA and LFS in this study facilitates simplifying the workflow for detecting VREFm, which is of great significance for rapidly detecting the enterococcal infections and preventing and controlling the nosocomial infections.

**IMPORTANCE:**

One of the key approaches to treating and controlling vancomycin-resistant *Enterococcus faecium* (VREFm) is an accurate and rapid diagnosis. To achieve this goal, a simple and rapid method must be constructed for immediate detection in the field. Multienzyme isothermal rapid amplification (MIRA) is an isothermal rapid amplification method that allows amplification reactions to be completed under room temperature conditions. When combined with lateral flow strips (LFSs), MIRA-LFS enables the rapid detection of pathogenic microorganisms. However, the MIRA method often produces false signals. These false signals are eliminated by using base mismatches introduced in primers and probes. The MIRA-LFS system was constructed with high specificity and sensitivity for the detection of VREfm, without the limitation of sophisticated instruments. This enables the prompt formulation of diagnostic and therapeutic decisions.

## INTRODUCTION

Enterococci are a ubiquitous group of gram-positive bacteria that are short-stranded, lack spores or pods, and can survive in harsh conditions. Enterococci are closely associated with human health and majorly cause healthcare-associated infections worldwide (1). Enterococci are commensal organisms of the human gastrointestinal tract, causing bacteremia, endocarditis, urinary tract infections, skin infections, and central nervous system infections (2). In addition, numerous research studies have shown that enterococci are the third most common cause of native valve endocarditis, after *Staphylococcus aureus* and *viridans* streptococci (1). Enterococci have a hard, thick cell wall and are inherently resistant to many antibiotics, and because of their highly “plastic” genome, they can develop acquired resistance through transposons on plasmids. Once a patient is infected with enterococci, the choice of therapeutic agents is limited (3). *Enterococcus faecalis* and *Enterococcus faecium* are the most common enterococci, with *E. faecium* having a broader spectrum of antibiotic resistance (4). In recent years, the extensive use of broad-spectrum antibiotics, especially vancomycin, has led to the emergence of vancomycin-resistant enterococci (VRE), especially vancomycin-resistant *Enterococcus faecium* (VREfm). VRE was first discovered and isolated in the UK and France in 1986. Subsequently, VRE infections were reported in the United States, Belgium, and Canada, spreading rapidly worldwide. Although the isolation rate of VRE in China is low, it is difficult to treat with a high mortality rate (5, 6). The VanA phenotype is the most prevalent in VREfm and has become globally disseminated.

In clinical laboratories, antimicrobial susceptibility tests and molecular biology assays are the principal methods for detecting VRE. The antimicrobial susceptibility test encompasses two distinct methodologies: the disc diffusion method and the minimum inhibitory concentration method (7). Both the Chinese expert consensus on VRE infection prevention and treatment and the American Association for Laboratory Standards recommend agar dilution as the standard test method (8). This method has a low error rate and is relatively inexpensive to perform. However, the agar dilution method necessitates a 24–48-h incubation period for the bacteria. It is no longer sufficiently timely to meet the demand for the early application of targeted antibiotics to treat VRE and reduce its high morbidity and mortality rate (7). Polymerase chain reaction (PCR) is the primary molecular biology technique for detecting VRE, offering rapidity and enhanced sensitivity, with the capacity to detect most VRE genotypes (9). Nevertheless, it is challenging to surmount laboratory constraints and fulfil the necessities of expeditious on-site testing. Consequently, there is a pressing need to identify a rapid, real-time, and accurate method for VRE.

Isothermal amplification technology provides novel approaches for nucleic acid amplification detection. Commonly used isothermal amplification techniques include Loop-mediated isothermal amplification (LAMP) and multienzyme isothermal rapid amplification (MIRA) (10). The LAMP method has been utilized for various pathogens, such as influenza and coronavirus (11, 12). However, compared with the LAMP method, MIRA is more convenient, adaptable to lower reaction temperatures, and has a more straightforward system (10). MIRA amplification system mainly includes the recombinase UvsX originating from the T4 phage, the single-stranded binding protein gp32, the coenzyme UvsY, and the DNA polymerase Bsu from *Bacillus subtilis*. In the presence of ATP, the recombinase UvsX binds to the primer with the assistance of coenzyme UvsY to form a recombinase-primer complex, which scans for homologous sequences on the target gene. This is followed by the formation of a D-loop structure. Concurrently, the DNA strand that has been replaced binds to gp32 to maintain the stability of the D-loop. Finally, UvsX was isolated, and DNA polymerase was bound to the 3′ end of the primer to amplify target genes (10). Currently, MIRA technology is employed to detect essential pathogens, including plague, anthrax, and Ebola (13, 14).

**FIG 1:**
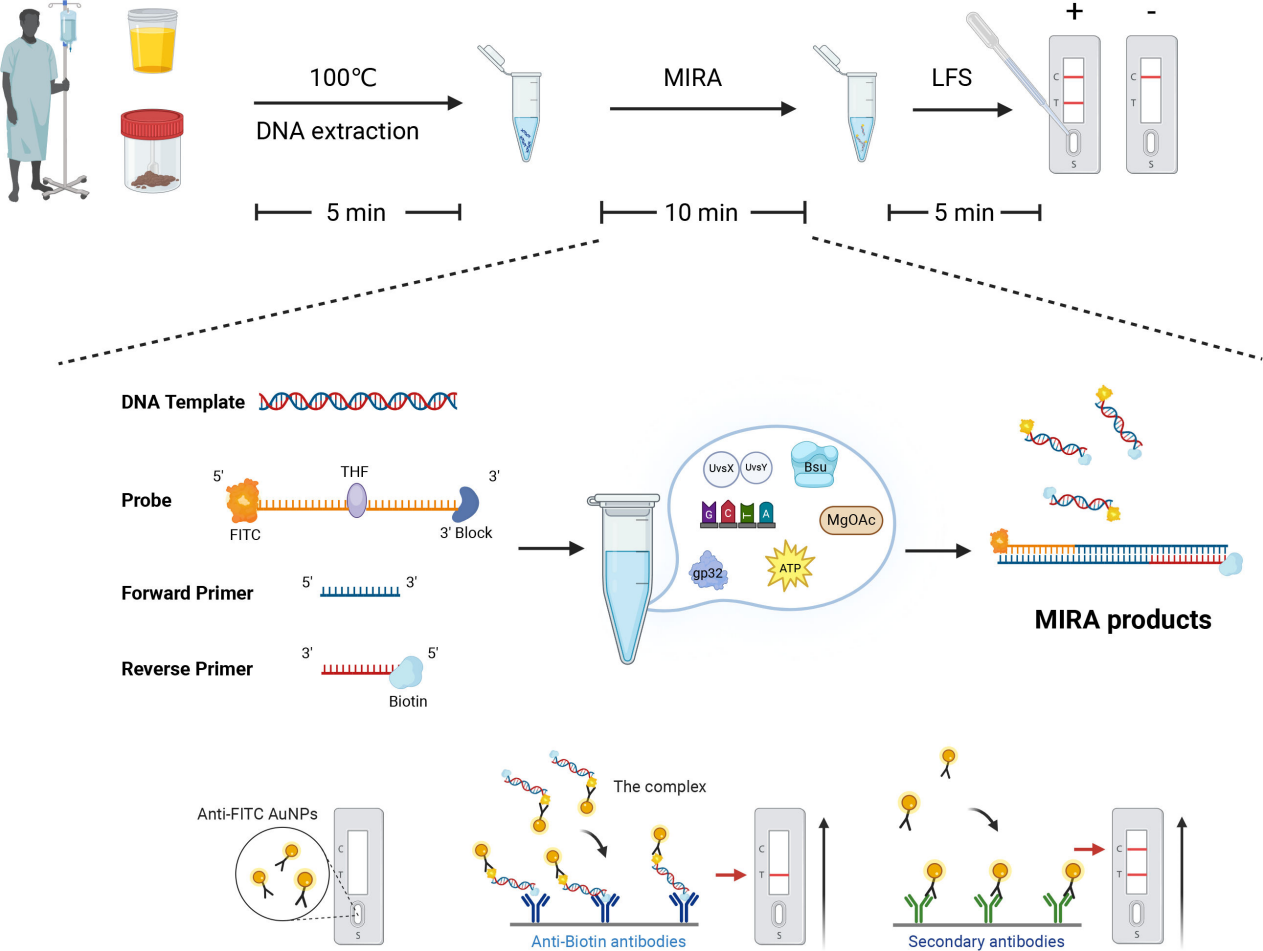
The principle of MIRA-LFS.

MIRA amplification products can be detected using agarose gel electrophoresis, probe-based fluorescence real-time system, and lateral flow strips (LFSs) (15). LFS is a paper-based device for point-of-care testing that uses gold nanoparticles (AuNPs) as a signal label (16). Combining MIRA with LFS technology can overcome laboratory limitations while maintaining the high specificity and high sensitivity of nucleic acid detection. More importantly, this method allows rapid amplification of target genes and visual detection of amplification products in a short period (17), reducing time and labor costs. The principle of MIRA-LFS is illustrated in Fig. 1.

The study aims to develop a rapid detection method for VREfm that is specific, sensitive, and real-time. This method will address the challenges of being time-consuming, having low sensitivity, and needing better specificity in detecting drug-resistant pathogens in clinical settings. It will also pave the way for more precise clinical diagnoses through advancements in laboratory medicine.

## MATERIALS AND METHODS

### Materials

#### Strain source

Clinical test specimens, including 37 fecal and 16 urine samples, were collected from January 2022 to December 2023 from The Second People’s Hospital of Lianyungang. The central laboratory provided VREfm (ATCC 51559) and other standard strains (Table 1).

**TABLE 1:** Other standard strains were used in this study

Strains	ATCC number
Methicillin-resistant *S. aureus*	ATCC 43300
*Acinetobacter baumannii*	ATCC 19606
*Streptococcus pneumoniae*	ATCC 49619
*Escherichia coli*	ATCC 25922
*Pseudomonas aeruginosa*	ATCC 27853
*Stenotrophomonas maltophilia*	ATCC 17666
*Staphylococcus haemolyticus*	ATCC 29970
*E. faecium*	ATCC 35667
*E. faecalis*	ATCC 29212
*Alcaligenes faecalis*	ATCC 31555

#### Main instruments and reagents

Microbial incubator (BluePard, China), Shaking incubator (Shanghai Minquan, China), Ultra-micro spectrophotometer (Quawell, USA), Electrothermal thermostatic water bath (Shanghai Jinghong, China), Electrophoresis apparatus (Bio-Rad, USA), Automated gel imaging analysis system (Shanghai Peiqing, China), DNA isothermal rapid amplification kit (#WLB8201KIT, AMP-Future Biotech Co., Ltd., China), nfo-DNA isothermal rapid amplification kit (#WLN8203KIT, AMP-Future Biotech Co., Ltd.), LFSs (#WLFS8201, AMP-Future Biotech Co., Ltd.).

### Methods

#### Microbial culture and sample pretreatment

The strains were cultured on Columbia Blood Agar, and a few colonies were selected and suspended in 100 µL of Tris-EDTA, which was then boiled at 100℃ for 30 min. The supernatant was centrifuged at 12,000 rpm for 1 min and stored at −20℃.

#### Primer and probe design

The MIRA amplification reaction requires a pair of forward and reverse primers and a probe. Primers and the probe for the MIRA reaction were designed using Primer Premier 5.0 software based on the gene sequence of VanA (GenBank: JN207930.1). The forward and reverse primers are 30–35 bp in size, and the probe is 46–53 bp in size. The reverse primer and probe are modified with biotin and fluorescein isothiocyanate (FITC) at 5′ ends, respectively. The probe was hindered at the 3′ end by a C3-spacer, and a single base in the center of the probe was substituted with tetrahydrofuran (THF). There are at least 30 bases preceding the THF site and at least 15 bases following the THF site. The MIRA reaction system consisted of a total of 50 µL: 29.5 µL of reaction buffer, 2.4 µL 10 µM of forward primer, 2.4 µL 10 µM reverse primer, 13.2 µL genomic template, and 2.5 µL 280 nM MgOAc. Each component was sequentially added to the reaction tube containing the enzyme component and thoroughly mixed. After transient centrifugation, the reaction mixture was incubated in a 37°C water bath for 30 min. At the end of the reaction, the MIRA amplification products were extracted using an equal volume of phenol-chloroform-isoamyl alcohol (25:24:1) mixture. Centrifuge at 12,000 rpm for 5 min. The appropriate amount of DNA sample buffer was added to the supernatant, and the reaction products were observed by 2% agarose gel electrophoresis.

#### Modification of primer and probe

When designing MIRA-LFS primers and probes, it is crucial to inhibit primer dimers to avoid false-positive signals completely. The probe and reverse primer were analyzed for cross-dimer formation in Primer Premier 5.0, and mismatches were performed. The principles for introducing mismatches were as follows: (i) eliminate the formation of a cross dimer by three consecutive bases at the 3′ end of the reverse primer, which is complementary to the probe; (ii) eliminate the cross-dimer formed by three bases on both sides of the THF site of the probe and the reverse primer; (iii) eliminate cross-dimers formed by more than three consecutive bases complementary to the primer and the probe. To ensure the amplification efficiency of the primer-probe set, avoid the mismatch of adjacent bases on both sides of the THF site of the probe and the mismatch of two consecutive bases.

#### Determine the optimal reaction temperature and time

The MIRA reaction system comprises a range of enzymes, and the efficiency of which is contingent on the time and temperature. Therefore, it is paramount to ascertain the most effective reaction time and temperature. The MIRA-LFS reaction system utilized a total volume of 50 µL, comprising 29.4 µL of reaction solution, 2 µL of 10 µM forward primer, 2 µL of 10 µM reverse primer, 0.6 µL of 10 µM probe, 13.5 µL of genome template and 2.5 µL of 280 nM MgOAc. The components were added sequentially to the reaction tube and thoroughly mixed. Subsequently, the reaction tube was subjected to an instantaneous centrifugation process, which was then incubated in a water bath. The reaction was conducted at 37°C, and the optimal time was determined by testing intervals of 0, 2, 4, 6, 8, 10, and 12 min. The optimal reaction temperature was determined by controlling the reaction time to 10 min and performing the amplification reaction with a temperature gradient of 28℃, 31℃, 34℃, 37℃, 40℃, 43℃, and 46℃, respectively. At the end of the reaction, 10 µL of the reaction product was diluted 20-fold with double-distilled water, and 50 µL of the diluted solution was added dropwise to the sample wells of the flow strip and waited for the color. The reaction conditions were established based on the intensity of the color produced.

#### Evaluate the specificity of MIRA-LFS technology for the detection of VREfm

MIRA-LFS detection was conducted on 10 other standard strains and 10 clinical sources of VREfm. The LFS detection results verified the system’s specificity.

#### Assess the assay sensitivity of the MIRA-LFS technique for the detection of VREfm

MIRA-LFS was carried out using VREfm genomic templates at concentrations ranging from 10^5^ to 10^−1^ CFU/µL. Ten independent experiments were conducted for each concentrations set. Probit regression analyses were performed based on the data from the 10 independent assays to determine the limit of detection (LOD).

#### Application evaluation of MIRA-LFS technology in clinical specimen examination

Clinical samples were tested using the MIRA-LFS method, agar dilution method, and PCR method, respectively. The compliance rate of the MIRA-LFS method compared to the other two methods was calculated by applying the following formula: (number of co-positive samples + number of co-negative samples) ÷ total number of samples × 100%.

## RESULTS

### Primer and probe design

**FIG 2:**
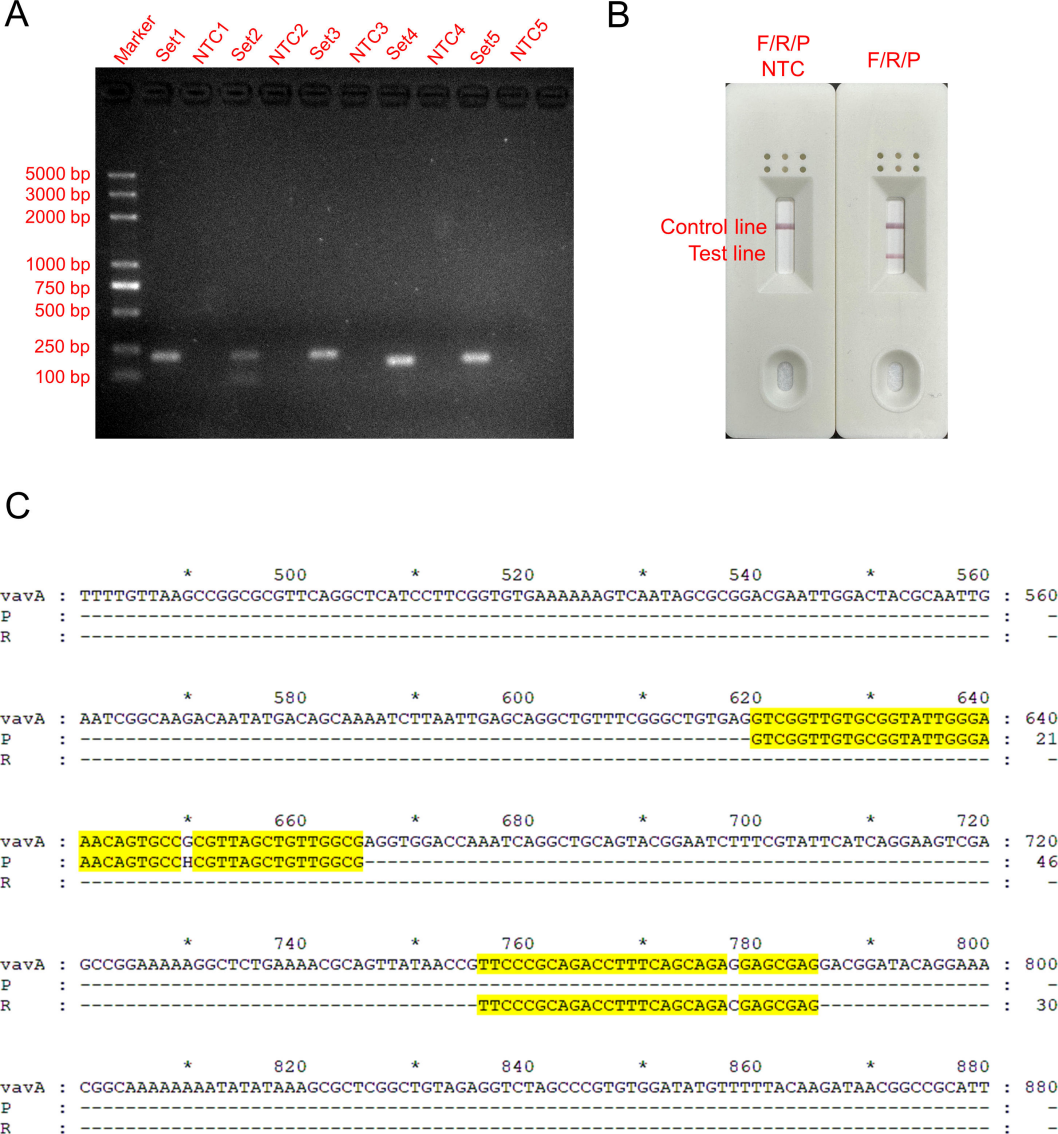
Selection of the best primer-probe pair. (A) Agarose gel electrophoresis results of basic MIRA products. (B) MIRA-LFS results after base mismatch. (C) Sequence alignment between the VanA gene and the F/R/P.

We designed five sets of primer pairs according to the conserved sequence of the VanA gene (Table 2). The optimal primer pairs were screened by basic isothermal amplification reaction and 2% agarose gel electrophoresis. As shown in Fig. 2A, the five sets of primer pairs produced bands of consistent size, following the anticipated outcomes. However, primer pair 2 exhibited discernible by-products. The No Template Control (NTC) groups demonstrated the absence of band formation. Primer pair 4 was the brightest one and did not display any visible primer dimers. Consequently, we selected primer pair 4 for probe design.

**TABLE 2:** Primer and probe sequences^*a*^

Primer set	Name	Sequence (5′−3′)	Length (bp)	Amplification product length (bp)
Set 1	F1	GGAGCGAGGACGGATACAGGAAACGGCAAA	30	212
	R1	CGAGCAAGCGGTCAATCAGGTCGGGAAGTG	30	
Set 2	F2	AGGCTGTTTCGGGCTGTGTGGTCGGTTGTG	30	208
	R2	TTTGCCGTTTCCTGTATCCGTCCTCGCTCC	30	
Set 3	F3	GGAGCGAGGACGGATACAGGAAACGGCAAA	30	214
	R3	TACGAGCAAGCGGTCAATCAGTTAGGGAAG	30	
Set 4	F4	GTCGGTTGTGCGGTATTGGGAAACAGTGCC	30	166
	R4	CTCGCTCGTCTGCTGAAAGGTCTGCGGGAA	30	
Set 5	F5	GTCGGTTGTGCGGTATTGGGAAACAGTGCC	30	176
	R5	TGTATCCGTGCTCGCTCGTCTGCTGAAAGG	30	
P		FITC-GTCGGTTGTGCGGTATTGGGAAACAGTGCC[H]CGTTAGCTGTTGGCG-/C3-Spacer/	45	214
R		Biotin-CTCGCTCGTCTGCTGAAAGGTCTGCGGGAA	30	
F		GTCGGTTGTGCGGTATTGGGAAACAGTGCC	30	

^*a*^ F, forward primer; R, reverse primer; P, probe; underlining indicates base mismatch site.

The probe was designed based on the forward primer of primer pair 4. The amplification performance of the primer-probe combination and the presence of false positives were detected by MIRA-LFS. The results are displayed in Fig. 2B. Following the introduction of appropriate base mismatches, the F/R/P set produced red bands on both the LFS detection and control lines, indicating that the F/R/P set exhibited good amplification performance. In the NTC group, no red bands were observed on the detection line, indicating no false-positive amplification of the F/R/P set after introducing the mismatch. The sequence alignment between the VanA gene of VREFm and the F/R/P is shown in Fig. 2C. Further experiments will be conducted utilizing the F/R/P set.

### Optimization of reaction conditions in the MIRA-LFS system

**FIG 3:**
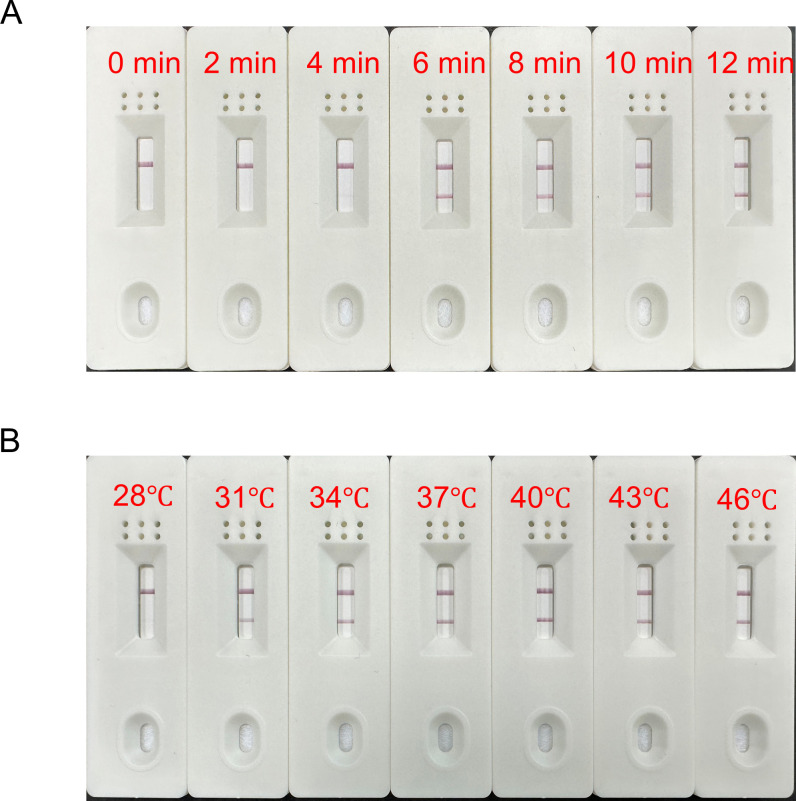
Optimization of MIRA-LFS reaction conditions. (A) The MIRA assay was optimized for reaction time. (B) The MIRA assay was optimized for reaction temperature.

We adjusted the reaction time and temperature to optimize the system’s reaction conditions. Firstly, the reaction temperature was controlled to be 37°C (Fig. 3A). The results indicated a faint band appeared in the detection line at 4 min, followed by a distinct and bright band at 6 min. After 8 min, there was no obvious change in the detection line with the extension of the reaction time. Next, the reaction time was controlled to be 10 min, and as shown in Fig. 3B, a lighter band appeared at a temperature of 31℃; the band was clear and bright in the range of 34−46℃. The optimal conditions for the MIRA-LFS reaction were 37℃ for 6 min, and the subsequent experiments were carried out under these conditions.

### Specificity of the MIRA-LFS system

**FIG 4:**
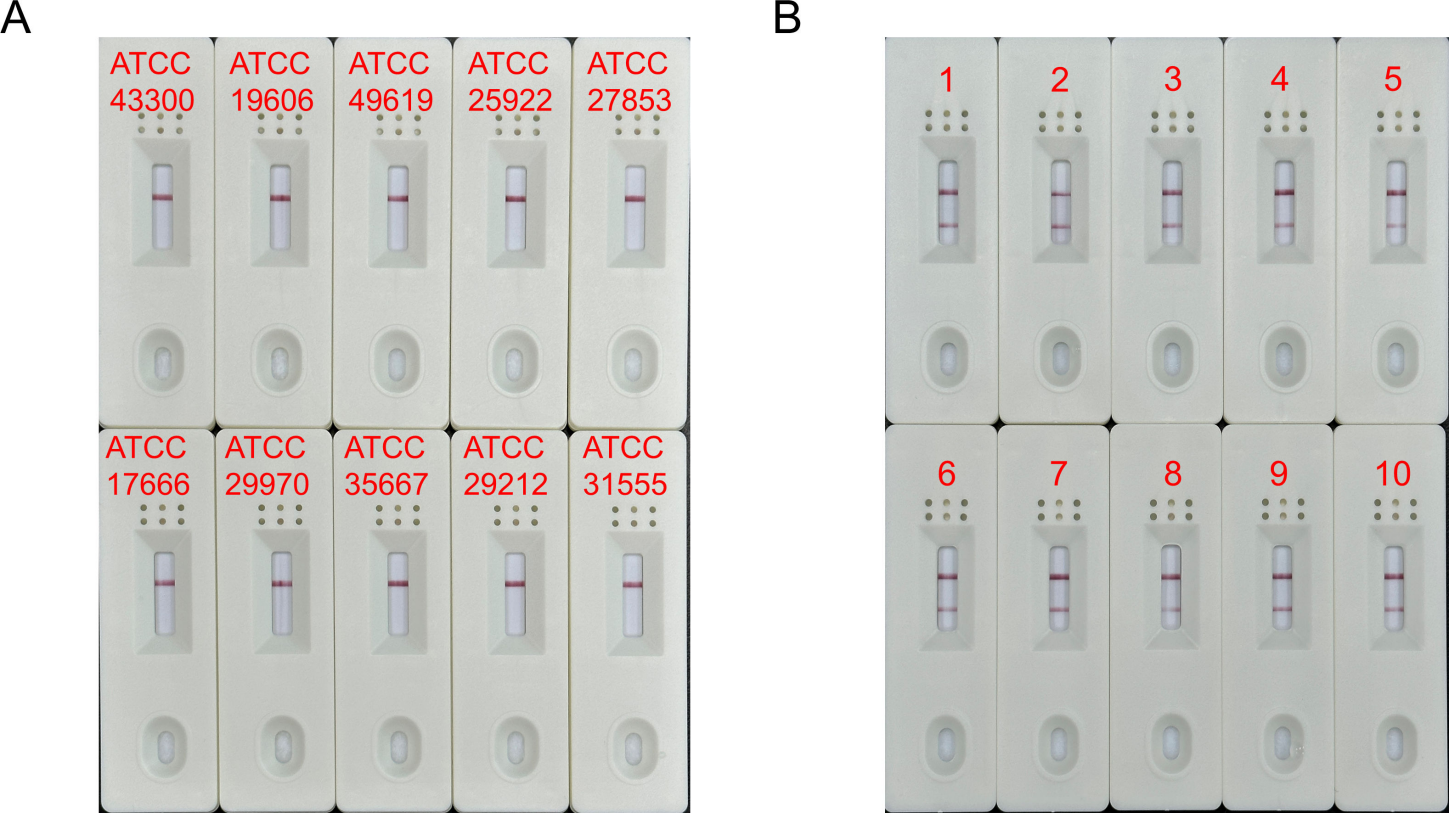
Specificity of the MIRA-LFS assay. (A) The MIRA-LFS assay was conducted using 10 standard pathogenic microbial strains. The ATCC IDs were labeled on the LFS. (B) The MIRA-LFS assay was conducted using 10 clinical VREfm. The sample numbers were labeled on the LFS.

In Fig. 4, no bands appeared on the LFS detection line when other standard strains were used as templates. However, a clear positive signal was observed on the detection line when the clinically sourced VREfmg was used as a template.

### Assay sensitivity of the MIRA-LFS system

**FIG 5:**
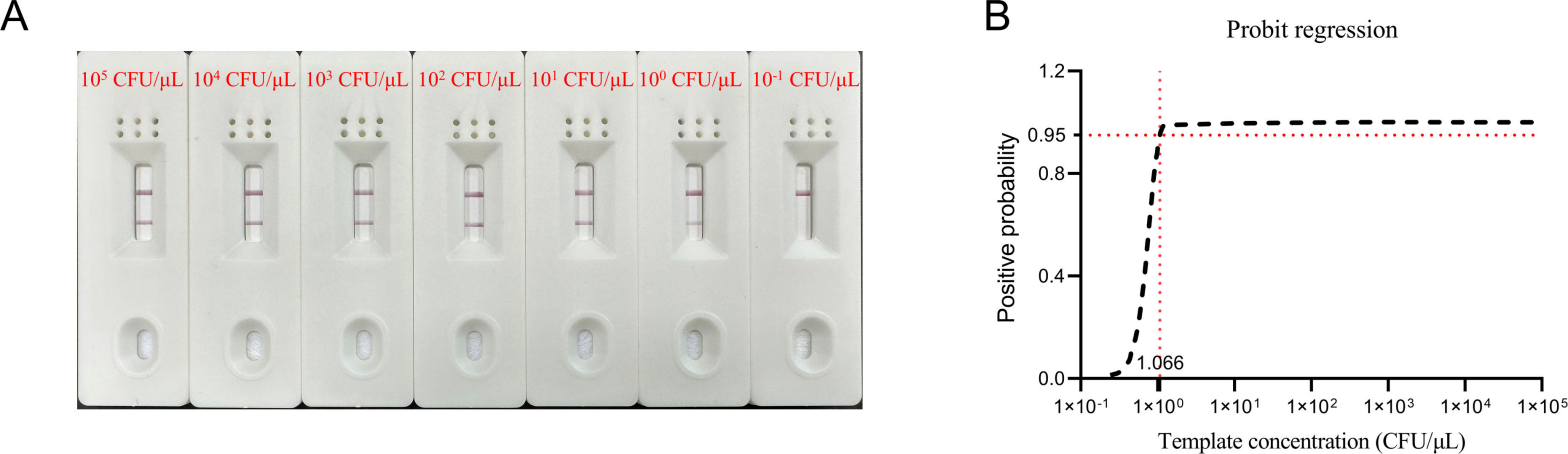
Assay sensitivity of the MIRA-LFS system. (A) The MIRA-LFS assay was conducted using different concentrations of VRFfm. The concentrations were labeled on the LFS. (B) The LOD of the MIRA-LFS was estimated using a probit regression model based on 10 independent replicates of each serial dilution.

The gradient dilution of the VREfm genome (10^5^, 10^4^, 10^3^, 10^2^, 10^1^, 10^0^, 10^−1^ CFU/µL) was employed as a template. As shown in Fig. 5A, the color of the positive bands weakened as the template concentration decreased. When the reaction system contained 10^−1^ CFU/µL of the genome, no bands were produced on the detection line. Furthermore, 10 independent assays were performed for all concentrations to ascertain the precise LOD of the MIRA-LFS assay. The results from the 10 assays were analyzed using probit regression in the SPSS software (Fig. 5B). The LOD for the MIRA-LFS reaction was 1.066 CFU/µL.

### Application of the MIRA-LFS system to clinical samples

The practical application value of the MIRA-LFS method in the clinic was evaluated by comparing it with PCR and agar dilution methods. Fifty-three collected clinical specimens were tested, and the results showed that the compliance rate of MIRA-LFS with the other two methods was 100% (Table 3), indicating that the system has good prospects for clinical application.

**TABLE 3:** Assay performance of different methods

MIRA-LFS	Agar dilution method/PCR			Accuracy rate
	Positive	Negative	Total	
Positive	10	0	10	100%
Negative	0	43	43	
Total	10	43	53	

## DISCUSSION

In this study, we employed the conserved sequence of the VanA gene of VREfm as a template to design the MIRA primers and probe. Because LFS recognizes FITC- and biotin-labelled sequences, we introduced base mismatches to avoid false-positive signals during the design of the probe and primers. By adjusting the time and temperature, we found that the optimal reaction conditions for this system were 6 min and 37°C. Subsequently, the sensitivity and specificity of the system were evaluated. MIRA-LFS was performed with 10^5^–10^−1^ CFU/µL of VREfm genomic DNA, respectively, and probit regression analysis was conducted, which demonstrated that the lowest detection limit of the system for VREfm was 1.066 CFU/µL. Finally, the system was tested on clinically sourced specimens to validate its clinical applicability. The results demonstrated that the established method was in complete agreement with those obtained using the PCR and agar dilution methods. This indicates that the method has good prospects for application.

VREfm was first reported in 1988 and has since spread rapidly throughout the United States, Europe, and elsewhere, with a significant increase in global prevalence (18). Statistical data indicate that the prevalence of VREfm in Europe has increased from 8.1% in 2012 to 19.0% in 2018 (19). VREfm, as a leading cause of healthcare-associated infections, poses a serious threat to public health safety. In 2017, the World Health Organization listed it as a global priority list of antibiotic-resistant bacterial pathogens (20). In recent years, the incidence of VRE infections in China has increased, with VRE becoming one of the most important pathogens associated with nosocomial infections. This emergence has presented new challenges to clinical microbiology and epidemiology. Consequently, the urgent need to develop rapid and accurate methods for detecting and identifying effective treatments has become apparent.

Vancomycin resistance arises due to replacing the C-terminal D-alanine residue in the precursor structure encoded by the manipulator by D-lactate or D-serine. This reduces the affinity of the original host-produced precursor for vancomycin and eliminates the target site for binding to vancomycin (21). Currently, there are nine resistance genotypes of VRE: VanA, VanB, VanC, VanD, VanE, VanG, VanL, VanM, and VanN. VanA and VanB are the most clinically significant (22). The genomes of the VanA and VanB are highly similar, and both were acquired from genetic determinants of resistance carried on the transposon Tn1546. However, VanA exhibits high levels of resistance to vancomycin and ticlopidine, whereas VanB is resistant to vancomycin and sensitive to ticlopidine (23, 24). The VanA phenotype is the most prevalent among the glycopeptide antibiotic-resistant enterococcal resistance genotypes and is the only one that can be detected in *S. aureus*. Consequently, infection control of the VanA genotype is of practical clinical significance.

Overall, the MIRA-LFS method, as a rapid, simple, and sensitive diagnostic method, helps simplify the complex workflow of detecting VREfm and is of great significance for the rapid detection of enterococcal infections as well as for the prevention and control of nosocomial infections. The method employs standard equipment and easy to disseminate, and the research results are easy to technologically translate, which has important social and economic effects.

## Data Availability

The data sets generated and/or analyzed during the current study are available from the corresponding author on reasonable request.

## References

[1] García-Solache M, Rice LB. 2019. The enterococcus: a model of adaptability to its environment. Clin Microbiol Rev 32:e00058-18. doi:10.1128/CMR.00058-18 30700430 PMC6431128

[2] O’Driscoll T, Crank CW. 2015. Vancomycin-resistant enterococcal infections: epidemiology, clinical manifestations, and optimal management. Infect Drug Resist 8:217–230. doi:10.2147/IDR.S54125 26244026 PMC4521680

[3] Cetinkaya Y, Falk P, Mayhall CG. 2000. Vancomycin-resistant enterococci. Clin Microbiol Rev 13:686–707. doi:10.1128/CMR.13.4.686 11023964 PMC88957

[4] Arias CA, Murray BE. 2012. The rise of the Enterococcus: beyond vancomycin resistance. Nat Rev Microbiol 10:266–278. doi:10.1038/nrmicro2761 22421879 PMC3621121

[5] Emaneini M, Hosseinkhani F, Jabalameli F, Nasiri MJ, Dadashi M, Pouriran R, Beigverdi R. 2016. Prevalence of vancomycin-resistant Enterococcus in Iran: a systematic review and meta-analysis. Eur J Clin Microbiol Infect Dis 35:1387–1392. doi:10.1007/s10096-016-2702-0 27344575

[6] Bonten MJ, Willems R, Weinstein RA. 2001. Vancomycin-resistant enterococci: why are they here, and where do they come from? Lancet Infect Dis 1:314–325. doi:10.1016/S1473-3099(01)00145-1 11871804

[7] Balouiri M, Sadiki M, Ibnsouda SK. 2016. Methods for in vitro evaluating antimicrobial activity: a review. J Pharm Anal 6:71–79. doi:10.1016/j.jpha.2015.11.005 29403965 PMC5762448

[8] Zakaria ND, Hamzah HH, Salih IL, Balakrishnan V, Abdul Razak K. 2023. A review of detection methods for vancomycin-resistant enterococci (VRE) genes: from conventional approaches to potentially electrochemical dna biosensors. Biosensors (Basel) 13:294. doi:10.3390/bios13020294 36832060 PMC9954664

[9] Satake S, Clark N, Rimland D, Nolte FS, Tenover FC. 1997. Detection of vancomycin-resistant enterococci in fecal samples by PCR. J Clin Microbiol 35:2325–2330. doi:10.1128/jcm.35.9.2325-2330.1997 9276411 PMC229963

[10] Tan M, Liao C, Liang L, Yi X, Zhou Z, Wei G. 2022. Recent advances in recombinase polymerase amplification: principle, advantages, disadvantages and applications. Front Cell Infect Microbiol 12:1019071. doi:10.3389/fcimb.2022.1019071 36519130 PMC9742450

[11] Imai M, Ninomiya A, Minekawa H, Notomi T, Ishizaki T, Tashiro M, Odagiri T. 2006. Development of H5-RT-LAMP (loop-mediated isothermal amplification) system for rapid diagnosis of H5 avian influenza virus infection. Vaccine (Auckl) 24:6679–6682. doi:10.1016/j.vaccine.2006.05.046 16797110

[12] Chaouch M. 2021. Loop-mediated isothermal amplification (LAMP): an effective molecular point-of-care technique for the rapid diagnosis of coronavirus SARS-CoV-2. Rev Med Virol 31:e2215. doi:10.1002/rmv.2215 33476080 PMC7995099

[13] Euler M, Wang Y, Heidenreich D, Patel P, Strohmeier O, Hakenberg S, Niedrig M, Hufert FT, Weidmann M. 2013. Development of a panel of recombinase polymerase amplification assays for detection of biothreat agents. J Clin Microbiol 51:1110–1117. doi:10.1128/JCM.02704-12 23345286 PMC3666764

[14] Huang P, Jin H, Zhao Y, Li E, Yan F, Chi H, Wang Q, Han Q, Mo R, Song Y, Bi J, Jiao C, Li W, He H, Wang H, Ma A, Feng N, Wang J, Wang T, Yang S, Gao Y, Xia X, Wang H. 2021. Nucleic acid visualization assay for middle east respiratory syndrome coronavirus (MERS-CoV) by targeting the UpE and N gene. PLoS Negl Trop Dis 15:e0009227. doi:10.1371/journal.pntd.0009227 33647020 PMC7951983

[15] Kim NK, Lee HJ, Kim SM, Jeong RD. 2022. Rapid and visual detection of barley yellow dwarf virus by reverse transcription recombinase polymerase amplification with lateral flow strips. Plant Pathol J 38:159–166. doi:10.5423/PPJ.NT.01.2022.0009 35385920 PMC9343894

[16] Wang Z, Zhao J, Xu X, Guo L, Xu L, Sun M, Hu S, Kuang H, Xu C, Li A. 2022. An overview for the nanoparticles-based quantitative lateral flow assay. S M 6:e2101143. doi:10.1002/smtd.202101143 35041285

[17] Zhao M, Wang X, Wang K, Li Y, Wang Y, Zhou P, Wang L, Zhu W. 2022. Recombinant polymerase amplification combined with lateral flow strips for the detection of deep-seated Candida krusei infections. Front Cell Infect Microbiol 12:958858. doi:10.3389/fcimb.2022.958858 36004333 PMC9394440

[18] Ye J-J, Shie S-S, Cheng C-W, Yang J-H, Huang P-Y, Wu T-S, Lee M-H, Huang C-T. 2018. Clinical characteristics and treatment outcomes of vancomycin-resistant Enterococcus faecium bacteremia. J Microbiol Immunol Infect 51:705–716. doi:10.1016/j.jmii.2017.08.025 29046248

[19] Ayobami O, Willrich N, Reuss A, Eckmanns T, Markwart R. 2020. The ongoing challenge of vancomycin-resistant Enterococcus faecium and Enterococcus faecalis in Europe: an epidemiological analysis of bloodstream infections. Emerg Microbes Infect 9:1180–1193. doi:10.1080/22221751.2020.1769500 32498615 PMC7448851

[20] Tacconelli E, Carrara E, Savoldi A, Harbarth S, Mendelson M, Monnet DL, Pulcini C, Kahlmeter G, Kluytmans J, Carmeli Y, Ouellette M, Outterson K, Patel J, Cavaleri M, Cox EM, Houchens CR, Grayson ML, Hansen P, Singh N, Theuretzbacher U, Magrini N, WHO Pathogens Priority List Working Group. 2018. Discovery, research, and development of new antibiotics: the WHO priority list of antibiotic-resistant bacteria and tuberculosis. Lancet Infect Dis 18:318–327. doi:10.1016/S1473-3099(17)30753-3 29276051

[21] Stogios PJ, Savchenko A. 2020. Molecular mechanisms of vancomycin resistance. Protein Sci 29:654–669. doi:10.1002/pro.3819 31899563 PMC7020976

[22] Tzavaras I, Siarkou VI, Zdragas A, Kotzamanidis C, Vafeas G, Bourtzi-Hatzopoulou E, Pournaras S, Sofianou D. 2012. Diversity of vanA-type vancomycin-resistant Enterococcus faecium isolated from broilers, poultry slaughterers and hospitalized humans in Greece. J Antimicrob Chemother 67:1811–1818. doi:10.1093/jac/dks166 22577103

[23] Chavers LS, Moser SA, Benjamin WH, Banks SE, Steinhauer JR, Smith AM, Johnson CN, Funkhouser E, Chavers LP, Stamm AM, Waites KB. 2003. Vancomycin-resistant enterococci: 15 years and counting. J Hosp Infect 53:159–171. doi:10.1053/jhin.2002.1375 12623315

[24] al-Obeid S, Collatz E, Gutmann L. 1990. Mechanism of resistance to vancomycin in Enterococcus faecium D366 and Enterococcus faecalis A256. Antimicrob Agents Chemother 34:252–256. doi:10.1128/AAC.34.2.252 2139314 PMC171567

